# Pre-operative Limbic System Functional Connectivity Distinguishes Responders From Non-responders to Surgical Treatment for Trigeminal Neuralgia

**DOI:** 10.3389/fneur.2021.716500

**Published:** 2021-10-04

**Authors:** Hayden Danyluk, Stefan Lang, Oury Monchi, Tejas Sankar

**Affiliations:** ^1^Division of Surgical Research, University of Alberta, Edmonton, AB, Canada; ^2^Division of Neurosurgery, Department of Surgery, University of Alberta, Edmonton, AB, Canada; ^3^Department of Clinical Neurosciences, University of Calgary, Calgary, AB, Canada; ^4^Health Research Innovation Centre, Hotchkiss Brain Institute, University of Calgary, Calgary, AB, Canada

**Keywords:** trigeminal neuralgia (TN), fMRI, limbic system, surgical response, treatment resistance

## Abstract

**Background:** Trigeminal neuralgia (TN) is a severe facial pain condition often requiring surgical treatment. Unfortunately, even technically successful surgery fails to achieve durable pain relief in many patients. The purpose of this study was to use resting-state functional magnetic resonance imaging (fMRI) to: (1) compare functional connectivity between limbic and accessory sensory networks in TN patients vs. healthy controls; and (2) determine if pre-operative variability in these networks can distinguish responders and non-responders to surgery for TN.

**Methods:** We prospectively recruited 22 medically refractory classic or idiopathic TN patients undergoing surgical treatment over a 3-year period, and 19 age- and sex-matched healthy control subjects. fMRI was acquired within the month prior to surgery for all TN patients and at any time during the study period for controls. Functional connectivity analysis was restricted to six pain-relevant brain regions selected *a priori*: anterior cingulate cortex (ACC), posterior cingulate cortex, hippocampus, amygdala, thalamus, and insula. Two comparisons were performed: (1) TN vs. controls; and (2) responders vs. non-responders to surgical treatment for TN. Functional connectivity was assessed with a two-sample *t*-test, using a statistical significance threshold of *p* < 0.050 with false discovery rate (FDR) correction for multiple comparisons.

**Results:** Pre-operative functional connectivity was increased in TN patients compared to controls between the right insular cortex and both the left thalamus [*t*_(39)_ = 3.67, *p* = 0.0007] and right thalamus [*t*_(39)_ = 3.22, *p* = 0.0026]. TN patients who were non-responders to surgery displayed increased functional connectivity between limbic structures, including between the left and right hippocampus [*t*_(18)_ = 2.85, *p* = 0.0106], and decreased functional connectivity between the ACC and both the left amygdala [*t*_(18)_ = 2.94, *p* = 0.0087] and right hippocampus [*t*_(18)_ = 3.20, *p* = 0.0049]. Across all TN patients, duration of illness was negatively correlated with connectivity between the ACC and left amygdala (*r*^2^ = 0.34, *p* = 0.00437) as well as the ACC and right hippocampus (*r*^2^ = 0.21, *p* = 0.0318).

**Conclusions:** TN patients show significant functional connectivity abnormalities in sensory-salience regions. However, variations in the strength of functional connectivity in limbic networks may explain why some TN patients fail to respond adequately to surgery.

## Introduction

Trigeminal neuralgia (TN) is a chronic, neuropathic facial pain disorder characterized by intermittent, typically unilateral, electric shock-like or stabbing pain attacks in the distribution of one or more branches of the trigeminal nerve (cranial nerve V—CNV) (1). TN is severely disabling, often fails to respond over the long-term to medications against neuropathic pain, and historically has been associated with a high suicide rate (2). A variety of surgical treatment options are available for medically refractory TN patients—including microvascular decompression (MVD), percutaneous rhizotomy, and stereotactic radiosurgery—but technically successful surgical treatment does not result in durable pain relief in many cases (2, 3). Even following MVD—clearly the most efficacious surgical treatment for TN—pain recurrence occurs in > 25% of patients within 2 years of surgery, followed by a 4% per year recurrence rate thereafter (4). Thus, there is a need to better understand the mechanisms underlying durable response to surgery in patients with TN.

Many cases of TN are associated with vascular compression affecting the root entry zone (REZ) of CNV (so-called *classical TN*) (1), and as a result a primary focus in TN research has been the structure of CNV studied using magnetic resonance imaging (MRI), in particular diffusion tensor imaging (DTI) (5, 6). However, a nerve-centric conceptualization of TN inadequately explains many key features of the disease, notably the development of medication-refractoriness and variability in response to treatment (4). Several structural and functional brain abnormalities have been identified in TN patients, particularly within the limbic system and closely connected paralimbic or sensory-salience structures [e.g., anterior cingulate cortex (ACC), insula, thalamus, and hippocampus (7–13)]. Brain abnormalities in TN show overlap with those observed in other chronic pain and headache conditions: in particular, altered resting-state functional connectivity and atrophy of limbic system structures are recurrent observations (7, 8, 10, 14–17), as are alterations in functional connectivity of the right insula, exemplified in migraine (18) and temporomandibular joint pain (19). How structural and functional brain alterations relate specifically to treatment-resistance in TN, however, has been relatively understudied. We recently showed that structural variability in the limbic system—specifically in hippocampal volume—may predict durability of pain relief following surgical treatment in TN (9). However, to date, functional MRI (fMRI) studies explicitly comparing functional connectivity between responders and non-responders to surgery are lacking.

Our central hypothesis was that pre-operative functional connectivity differences exist between responders and non-responders to surgical treatment for TN. Our primary objective was to perform a focused functional connectivity analysis in TN patients and healthy control (HC) subjects, first identifying key networks which are altered in TN. We then evaluated how functional connectivity within these networks related to surgical outcome. Our analysis was restricted to six regions of interest (ROI) determined *a priori* (ACC, posterior cingulate cortex (PCC), hippocampus, amygdala, thalamus, and insula) that are part of previously characterized acute (sensory-salience related) or chronic (emotion-related) pain activity patterns (20, 21). In addition to examining pre-operative functional connectivity differences between eventual responders and non-responders, we further examined how functional connectivity within our selected ROIs correlated with time since initial TN diagnosis, given that surgical non-response has been linked to longer duration of TN (22).

## Methods

### Study Participants

This was a single-center, prospective, longitudinal study of patients undergoing surgical treatment for TN between 2017 and 2020. This study was approved and performed in accordance with the rules and regulations by the Health Research Ethics Board—Health Panel of the University of Alberta. Potential study patients were identified in the neurosurgery clinic, then recruited by telephone. All participants provided written informed consent. *Inclusion criteria:* medically refractory classic or idiopathic TN defined using International Classification of Headache Disorders-III (ICHD-III) criteria (1); scheduled for surgical treatment by MVD or percutaneous balloon compression rhizotomy (BC). *Exclusion criteria:* history of multiple sclerosis or other lesional causes of TN; diagnosed psychiatric illness; history of any prior non-TN neurosurgical procedures. Additionally, we recruited 19 HC subjects matched to the TN group in mean age and sex distribution, and without chronic pain or psychiatric conditions.

### Data Acquisition

TN patients underwent MRI scanning within a one-month period prior to surgery, while HC subjects underwent a single MRI scanning session at any time during the study period. Scanning was carried out on a 3T Siemens Prisma Magnetom MRI scanner (Erlangen, Germany) with 64-channel head radiofrequency coil. Study participants underwent: *3D T1-weighted structural scan* [magnetization-prepared rapid acquisition gradient echo (MPRAGE)], field-of-view (FOV) = 250 × 250 mm^2^, 208 slices, 0.85 mm isotropic, repetition time (TR) = 1800 ms, echo time (TE) = 2.37 ms, inversion time (TI) = 900 ms, 8° flip angle, 3:41 min) and resting-state T2^*^ *functional MRI scan* (multiband gradient-echo echo-planar imaging sequence, FOV = 224 × 224 mm^2^, 60 slices, 2.2 mm isotropic, TR = 1,830 ms, TE= 30 ms, matrix = 102 × 102, 80° flip angle, volumes = 252, multiband acceleration factor = 2, parallel imaging factor = GRAPPA factor 2, phase encoding direction = anterior-posterior, Bandwidth = 2450 Hz/pixel, 8:02 min). During resting-state fMRI acquisition, participants were instructed to keep their eyes closed but not to fall asleep or focus on anything in particular. Additionally, prior to MRI scanning TN patients completed a pain questionnaire to report the severity of pain attacks over the past week using a 0-100 mm Visual Analog Scale (VAS), and to accurately describe the frequency and location of attacks. TN patients were followed for at least 12-months after surgery (see below for details).

### Clinical Characteristics and Outcome Assessment

The following demographic/clinical data were collected: sex; age; duration of TN since diagnosis; side-of-pain; pre-operative pain severity (measured using VAS); first (virgin) or repeat surgical treatment for TN; surgery type (MVD or BC); and medications (carbamazepine/oxcarbazepine (yes/no), gabapentin/pregabalin (yes/no), other antiepileptic (yes/no), antidepressant/anxiolytic (yes/no), baclofen (yes/no), opioid (yes/no), and cannabis oil (yes/no). Study participants were classified as responders or non-responders as follows: *responders*—(1) documented evidence of immediate and persistent pain relief for at least 1 year after surgery, as defined by a Barrow Neurological Institute (BNI) facial pain score (23) of 1, 2, or 3a; (2) no offer of or repeat surgical TN treatment within the 1 year following surgery; *non-responders*—(1) inadequate initial pain relief from surgery or early pain recurrence within 1 year of surgery, as defined by a BNI facial pain score of 3b, 4, or 5; or (2) offered or underwent repeat surgical treatment within 1 year of surgery. TN patients were followed-up longitudinally by in-person visits with study personnel at 7- and 30-days following surgery, and by phone follow-up at 6- and 12-months after surgery. BNI facial pain score was determined at each visit; any patient who had changed from an earlier post-operative BNI score of 1, 2, or 3a to 3b, 4, or 5, was immediately reclassified as a non-responder. Additionally, patients at a minimum underwent follow-up with their treating surgeon at 4-6 weeks post-operatively, and additional follow-up visits with treating surgeons thereafter were made on an *ad hoc* basis (usually because patients had developed recurrent pain).

### fMRI Analysis

#### Pre-processing

All subjects underwent standard pre-processing in *SPM12*, including realignment, slice-time correction, and segmentation into gray matter, white matter, and cerebrospinal fluid (CSF) components using SPM's Unified Segmentation (24). Images were directly (non-linearly) normalized to MNI space using an EPI template (25). De-noising was performed using *Conn* v18.a software (https://web.conn-toolbox.org) (26), which included regression of six movement parameters and their first temporal derivatives and implementation of CompCor by performing PCA on eroded white matter and CSF masks with regression of the first 5 components (27). Volumes with large (>0.9 mm) frame-wise displacement or global signal change [>5 standard deviations (SD)] were also included as covariates of no interest. Linear de-trending was performed to remove signal drift, while high frequency noise was excluded by subjecting the residual signal to a high pass filter (>0.008 Hz).

#### Functional Connectivity

**Figure 1:**
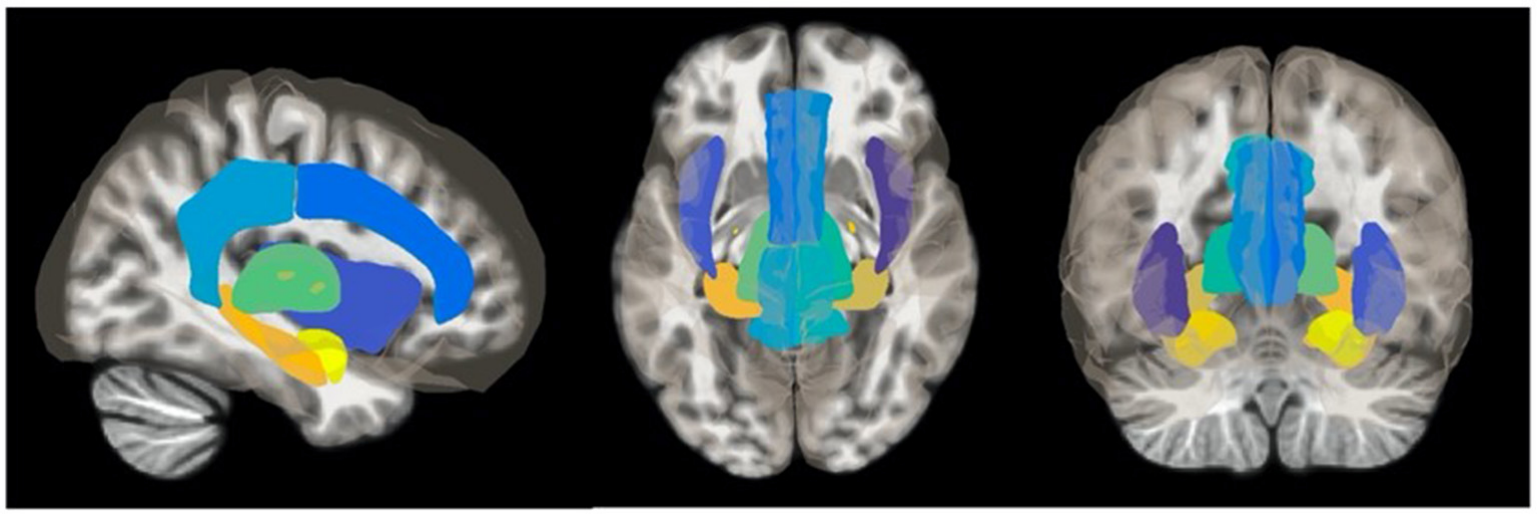
Regions of interest (ROIs) used as seed points in fMRI analyses. Resting-state fMRI analysis was restricted to 10 ROIs composed of bilateral limbic and accessory sensory structures determined *a priori*: insula, thalamus, amygdala, hippocampus, anterior cingulate cortex, and posterior cingulate cortex. ROIs were generated from the Harvard-Oxford Atlas (28).

Ten ROIs made up of limbic system and paralimbic structures were selected *a priori* from previously characterized acute- and chronic-pain activity patterns to be used as nodes for a focused functional connectivity analysis (21): bilateral insular cortex, bilateral amygdala, bilateral hippocampus, bilateral thalamus, anterior cingulate cortex, and posterior cingulate cortex (Figure 1). Each ROI was generated from the Harvard-Oxford Atlas (28). The residual BOLD time-course was averaged within each ROI, and functional connectivity between each node of the limbic system was calculated as the Fisher transformed Pearson correlation coefficient. Differences in the pairwise connectivity of each limbic node between HC and TN patients was assessed with a two-sample *t*-test, using a threshold for statistical significance of *p* < 0.050 with a false discovery rate (FDR) correction for 10 seeds (9 comparisons). This was repeated for each individual node. Similarly, functional connectivity between the same 10 ROIs was used to compare responders (*n* = 16) and non-responders (*n* = 6) to surgical treatment for TN. In this latter analysis, we adjusted for the influence of the side of TN pain and immediate pre-scan VAS pain severity by including these as covariates in an ANCOVA model. Pre-scan VAS score was included to mitigate the influence of acute pain state on connectivity differences. ROI pairs demonstrating connectivity differences between surgical outcome groups were subsequently correlated with duration of pain using Pearson correlation.

### Statistical Analysis

Clinical characteristics and demographic variables were compared between responders and non-responders to surgical treatment using the Mann-Whitney U test, as well as Chi-square or Fisher's exact test where appropriate. Statistical analyses were carried out with GraphPad Prism version 8 for Mac OS X (GraphPad Software, La Jolla California, USA). Statistical significance was set at *p* < 0.050 (2-tailed).

## Results

### Study Participants

Twenty-two TN patients and 19 HCs were included in this study between 2017 and 2020 (Table 1).

**Table 1:** Comparison of demographic and clinical characteristics between TN patients (including responders and non-responders) and healthy controls.

	**Responders**	**Non-Responders**	***P*** **-value (2-tailed)**	**TN**	**HC**	***P*** **-value (2-tailed)**
Group	16	6	–	22	19	–
Sex (Female/Male)	7/9	5/1	0.16	12/10	10/9	0.90
Age (years)	60.4 ± 9.7	53.0 ± 12.9	0.12	56.5 ± 10.9	55.4 ± 9.3	0.72
Duration of TN (years)	4.6 ± 3.3	10.5 ± 5.9	0.021^*^	6.2 ± 4.8	N/A	–
Side of pain (right/left)	10/6	4/2	>0.99	14/8	N/A	–
Pre-op VAS (mm)	79.9 ± 24.1	72.3 ± 37.2	0.84	72.8 ± 27.5	N/A	–
Virgin (yes/no)	14/2	3/3	0.10	17/5	N/A	–
Surgery type (MVD/BC)	12/4	3/3	0.33	15/7	N/A	–
Carbamazepine/oxcarbazepine (yes/no)	15/1	6/0	>0.99	21/1	N/A	–
Gabapentin/pregabalin (yes/no)	6/10	5/1	0.15	11/11	N/A	–
Other antiepileptics (yes/no)	2/14	1/5	>0.99	3/19	N/A	–
Antidepressant/anxiolytic (yes/no)	2/14	1/5	>0.99	3/19	N/A	–
Baclofen (yes/no)	2/14	4/2	0.025^*^	6/16	N/A	–
Opioid (yes/no)	0/16	1/5	0.27	1/21	N/A	–
Cannabis oil (yes/no)	1/15	1/5	0.48	2/20	N/A	–

Mann-Whitney, Chi-square, or Fishers-exact tests used where appropriate. Means **±** standard deviations are presented. Virgin (yes/no): first-time surgical treatment for TN; **MVD**: microvascular decompression surgery; **BC**: percutaneous balloon compression rhizotomy; other antiepileptics: lamotrigine, topiramate; antidepressant/anxiolytic: amitriptyline, duloxetine. Threshold for statistical significance set at ^*^ p < 0.05.

### Clinical Characteristics and Demographics

#### TN vs. HC Comparison

TN patients and HCs were well-matched in age (mean 56.5 ± 10.9 years and 55.4 ± 9.3 years respectively, *p* = 0.72) and sex distribution (12F/10M and 10F/9M, *p* = 0.90). Average duration of TN from diagnosis to surgery was 6.2 ± 4.8 years, with right-sided TN being more common than left-sided TN (14R/8L). Pre-operative VAS was 72.8 ± 27.5. Across TN patients, 15 underwent MVD and 7 underwent BC, with 17/22 undergoing virgin surgical treatments (14MVD, 3BC). All TN patients were on antiepileptic medication at the time of surgery, including carbamazepine/oxcarbazepine (*n* = 21) and/or gabapentin/pregabalin (*n* = 11). Three TN patients were also on antidepressant/anxiolytic medication, six were on baclofen, one was taking opioids, and two others were taking cannabis oil. Clinical characteristics and demographic features of all study participants are presented in Table 1.

#### Responders vs. Non-responders

In total, there were 16 responders to surgery and 6 non-responders. Most non-responders were female (5F/1M), while responders display a balanced sex distribution (7F/9M), though the difference was not statistically significant (*p* = 0.16). There was no difference in average age of responders and non-responders (60.4 ± 12.7 vs. 53.0 ± 12.9 years, *p* = 0.12). Non-responders had a longer duration of TN prior to surgical treatment than responders (10.5 ± 5.9 vs. 4.6 ± 3.3 years respectively, *p* = 0.021). Distribution of surgery type did not differ between outcome groups. The proportion of patients taking baclofen was higher in non-responders than responders (*p* = 0.025), while there were no other differences in medication use. Individual clinical profiles of TN patients are presented in Table 2, and post-operative variations in BNI facial pain score are indicated in Supplementary Figures 1, 2.

**Table 2:** Clinical characteristics of TN patients.

**Patient ID**	**Sex**	**Age (years)**	**Side**	**Duration of TN (years)**	**Pre-op VAS (mm)**	**Branch(es)**	**SX type**	**# prev. SX**	**BNI**	**Medications**
**Responders**										
1	M	57.5	R	6	66	3	MVD	0	1	Carbamazepine
2	M	49.0	R	1	100	1/2/3	BC	3	3a	Oxcarbazepine, baclofen
3	M	45.1	L	9	98	1	MVD	0	1	Carbamazepine, pregabalin
4	F	58.5	R	11	100	2/3	MVD	0	1	Carbamazepine
5	M	63.9	R	8	82	1/2/3	MVD	0	1	Carbamazepine
6	M	67.5	R	6	71	2/3	BC	0	3a	Carbamazepine
7	F	74.1	L	3	81	2	MVD	0	1	Oxcarbazepine, pregabalin
8	F	60.3	L	6	36	2/3	MVD	1	3a	Gabapentin, amitriptyline
9	F	64.9	L	7	100	2/3	MVD	0	1	Carbamazepine, gabapentin
10	F	60.4	L	7	86	2/3	MVD	0	3a	Carbamazepine, oxcarbazepine
11	F	60.4	R	1	80	2/3	BC	0	3a	Carbamazepine
12	M	41.8	R	2	89	1/2	MVD	0	1	Carbamazepine, gabapentin, topiramate
13	F	68.5	L	1	100	3	MVD	0	1	Carbamazepine
14	M	61.5	R	2.5	79	3	MVD	0	3a	Carbamazepine
15	M	63.3	R	2.5	95	2/3	MVD	0	1	Oxcarbazepine, lamotrigine, gabapentin
16	M	40.6	R	1	15	2/3	BC	0	2	Carbamazepine, baclofen, duloxetine, cannabis oil
**Non-responders**										
17	F	37.3	L	6	63	2/3	MVD	0	4	Oxcarbazepine, baclofen
18	F	48.9	L	8	2	1/2/3	MVD	0	5	Carbamazepine, gabapentin, baclofen
19	M	69.5	R	19	100	3	BC	1	4	Oxcarbazepine, gabapentin
20	F	57.1	R	14	80	1/2/3	BC	2	5	Carbamazepine, gabapentin, lamotrigine, baclofen, hydromorphone
21	F	57.5	R	13	100	1/2/3	BC	2	5	Carbamazepine, gabapentin
22	F	36.3	R	3	89	2/3	MVD	0	4	Carbamazepine, gabapentin, baclofen, amitriptyline, cannabis oil

***M**, male; **F**, female; **Side**, side of facial pain (**R**, right; **L**, left); **Branch(es)**: affected trigeminal nerve branches (i.e., V1, V2, V3, or combination); **SX**, surgical treatment; **MVD**, microvascular decompression; **BC**, percutaneous balloon compression rhizotomy; **# of prev. SX**, number of previous surgical treatments for TN; **BNI**, Barrow Neurological Institute facial pain intensity score*.

### Resting-State Connectivity Analyses

#### HC vs. TN

**Figure 2:**
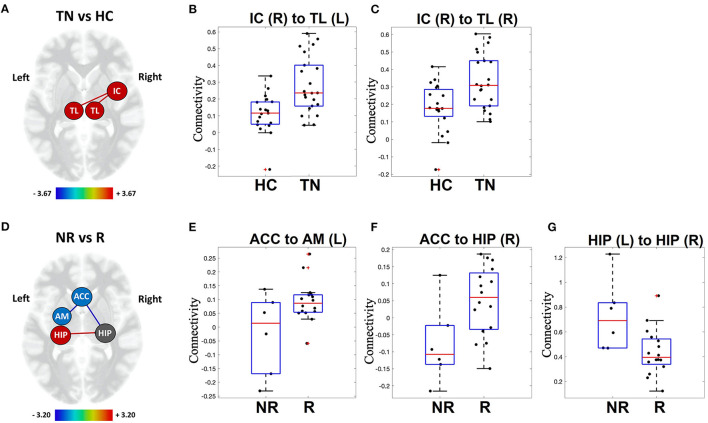
Visual representation of functional connectivity differences. *TN patients (TN) vs. healthy controls (HC)*: patients with TN show increased resting-state functional connectivity in sensory-relay and salience structures **(A)**. Individual patient connectivity is displayed on scatter-plot graphs for right insular cortex to left thalamus **(B)** and right insular cortex to right thalamus connections **(C)**. *Responders (R) vs. Non-responders (NR)*: non-responders show altered resting-state functional connectivity in limbic structures **(D)**. Individual patient connectivity is displayed on scatter-plot graphs for the anterior cingulate cortex to left amygdala **(E)** and right hippocampus **(F)**, as well as between the left and right hippocampus **(G)**. Increased connectivity, red line; decreased connectivity, blue line; TL, thalamus; IC, insular cortex; HIP, hippocampus; AM, amygdala; ACC, anterior cingulate cortex. Heat scales indicate the relative strength of connection between nodes (i.e., edge color). Red crosses represent patients with functional connectivity beyond 1.5 times the group inter-quartile range (IQR).

Resting-state functional connectivity was increased in TN patients between the right insular cortex and left thalamus [t_(39)_ = 3.67, *p* = 0.0007], as well as the right insular cortex and the right thalamus [*t*_(39)_ = 3.22, *p* = 0.0026]. *HC vs TN* connectivity results are presented in Table 3 and Figure 2.

**Table 3:** Functional connectivity differences between TN patients and healthy controls, and responders and non-responders to surgical treatment for TN.

**Connectivity difference**	**Seed region**	**Connection**	***T*** **-value**	***P*** **-value (raw)**	***P*** **-value (FDR adjusted)**
**TN vs. Healthy controls**					
Increased connectivity	Thalamus (left)	Insular cortex (right)	*t*_(39)_ = 3.67	*p* = 0.0007^*^	0.0065^*^
Increased connectivity	Thalamus (right)	Insular cortex (right)	*t*_(39)_ = 3.22	*p* = 0.0026^*^	0.0117^*^
**Non-responders vs. Responders**					
Increased connectivity	Hippocampus (left)	Hippocampus (right)	*t*_(18)_ = 2.85	*p* = 0.0106^*^	0.0477^*^
Decreased connectivity	ACC	Amygdala (left)	*t*_(18)_ = −2.94	*p* = 0.0087^*^	0.0392^*^
Decreased connectivity	ACC	Hippocampus (right)	*t*_(18)_ = −3.20	*p* = 0.0049^*^	0.0392^*^

TN patients vs. healthy controls: patients with TN show increased resting-state functional connectivity between the thalamus (both left and right) and right insula. Responders vs. Non-responders: non-responders show increased resting-state functional connectivity between the left and right hippocampus. Non-responders also show decreased resting-state functional connectivity between the anterior cingulate cortex and left amygdala and right hippocampus. Two-sample Student's t-test used with false-discovery rate (FDR) correction. Threshold for statistical significance was set at ^*^p < 0.05. ACC, anterior cingulate cortex.

#### Responders vs. Non-responders

Non-responders to surgical treatment for TN showed increased resting-state functional connectivity between the left and right hippocampus [*t*_(18)_ = 2.85, *p* = 0.0106] compared to responders. Non-responders also showed decreased resting-state functional connectivity between the ACC and left amygdala [*t*_(18)_ = −2.94, *p* = 0.0087], as well as the ACC and right hippocampus [*t*_(18)_ = −3.20, *p* = 0.0049]. *Responder vs. non-responder* connectivity results are presented in Table 3 and Figure 2 (raw data in Supplementary Table 1).

#### Duration of Illness and Functional Connectivity

**Figure 3:**
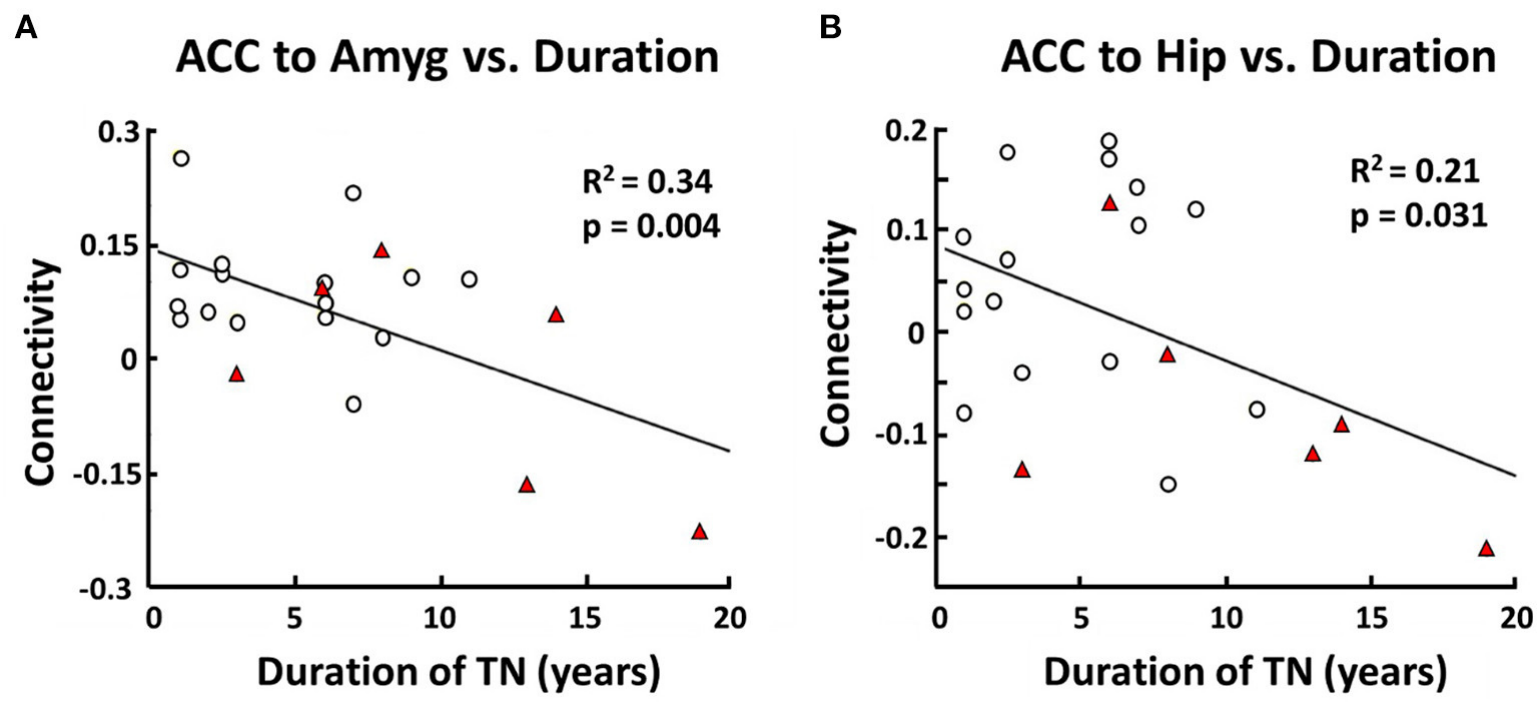
Correlation between functional connectivity (responders vs. non-responders) and duration of TN. In TN patients there is a negative correlation between anterior cingulate cortex (ACC) and left amygdala (Amyg) resting-state functional connectivity and duration of TN in years (*R*^2^ = 0.34, *p* = 0.004) **(A)**. There is also a negative correlation between ACC and right hippocampus (Hip) resting-state functional connectivity and duration of TN in years (*R*^2^ = 0.21, *p* = 0.031) **(B)**. Pearson Correlation was used with a threshold for statistical significance set at *p* < 0.05.

Based on the result that non-responders had characteristic differences in functional connectivity between three pairs of structures (i.e., ACC-left amygdala, ACC-right hippocampus, left hippocampus-right hippocampus), we examined whether the strength of functional connectivity for each of these pairs was related to duration of TN illness from the time of diagnosis. Indeed, across all TN patients, duration of illness was negatively correlated with connectivity between the ACC and the left amygdala (*r*^2^ = 0.34, *p* = 0.00437). Similarly, duration of illness was also negatively correlated with connectivity between the ACC and the right hippocampus (*r*^2^ = 0.21, *p* = 0.0318; Figure 3). However, there was no correlation between duration of illness and connectivity between the left and right hippocampus.

## Discussion

In this single-center prospective study, we used resting-state fMRI to analyse pre-operative functional connectivity—with a focus on sensory-salience and limbic networks—in patients with medically refractory TN. To our knowledge, this is the first direct comparison of functional connectivity prior to surgery between responders and non-responders to surgical treatment for TN. We observed increased functional connectivity between the bilateral thalamus and right insular cortex in TN patients compared to age- and sex-matched HC subjects, indicating that functional abnormalities in sensory-salience regions are present in TN. We also observed pre-operative functional connectivity differences between surgical responders and non-responders, though these differences were found within a network confined to the limbic system and included the ACC, amygdala, and hippocampus. Additionally, the magnitude of functional connectivity differences within this network of limbic structures was strongly correlated with duration of illness across all TN patients. Taken together, our results suggest that while functional abnormalities in sensory-salience structures characterize patients with TN, it may principally be variations in limbic network function that contribute to poor response to surgical treatment in TN.

Our patients were suitably representative of medically refractory TN sufferers who are offered surgery, and overall demonstrated a 73% surgical response rate at 1 year, in agreement with our previous work and the existing literature, notwithstanding differences in the categorization of surgical outcome across studies (4, 9). In line with previous reports, non-responders showed a female preponderance and on average suffered from TN for more than twice as long as responders at the time of surgery (22). All TN patients were taking antiepileptic medication, and medication use was largely the same between responders and non-responders at a medication-class level. However, it is worthwhile to note that 50% of responders were exclusively taking first-line medications (e.g., carbamazepine or oxcarbazepine), while all non-responders had progressed to second-line medication classes at the time of surgery, perhaps reflecting greater medical-refractoriness (3).

Compared to HC subjects, TN patients showed increased functional connectivity between the bilateral thalamus and right insula. These findings are in line with recent reports of thalamic hyperactivity compared to HCs both in TN (8) and in other pain conditions affecting the trigeminal system, such as migraine (29). The present study did not identify abnormal amygdala functional connectivity in TN patients. This contrasts with the findings of Zhang et al. (10), though it is possible that the discrepancy may be explained by different fMRI seed strategies used by their study compared to ours. We have previously reported that thalamus volume is enlarged in TN contralateral to the side-of-pain, which may reflect a structural consequence of sustained hyperactivity (9, 30). The present findings add support to the notion that abnormalities in sensory-relay architecture are indeed a robust feature in patients with TN, though it must be pointed out that thinking of the thalamus exclusively as a sensory-relay structure would be an oversimplification. While the insula certainly participates in sensory-relay, it also has a key role in higher-order functions such as salience and the redirection of attention/focus (16). The right insula in particular has been shown to have increased functional activity in TN (12) and acute pain states (16, 31), as well as reduced volume in patients with chronic pain conditions (7, 10, 15). Given the complementary functions of the thalamus and insula in sensory-relay and salience, respectively, and because these two structures have direct structural connections (32), we speculate that our findings suggest increased sensory load coming from, and therefore increased focus on, the painful region of the face in TN. It is impossible to know from our data whether abnormalities in the thalamus and insula are the cause or an effect of medically refractory TN. However, the previously reported findings that right insular structure normalizes following successful surgical treatment for TN supports the latter interpretation (7).

As mentioned above, this is first the study to our knowledge to compare pre-operative resting-state functional connectivity between responders and non-responders to surgical treatment for TN. Non-responders showed increased functional connectivity between the left and right hippocampus. Our observation that hippocampus activity is altered in non-responders overlaps with previous findings that hippocampus structure is associated with pain severity and durability of pain relief in TN (9, 33), and possibly that hippocampal neurogenesis may directly influence pain persistence (at least in animal models) (34). It is plausible that increased left-right hippocampal connectivity may reflect or contribute to an increased capacity for pain memory recall (35). We also observed that the ACC in non-responders showed decreased functional connectivity to both the left amygdala and right hippocampus. The pre-operative difference in this ACC-hippocampus-left amygdala network (i.e., a limbic network) between responders and non-responders suggests that limbic system contributions to the chronification of pain may also be relevant in treatment-resistant TN (17, 35, 36). Interestingly, functional connectivity within this limbic network correlates negatively with duration of illness. While functional connectivity in TN patients has previously been shown to correlate with duration of illness (8), this is the first time that variability in pre-operative network connectivity associated with duration of TN has been shown to correlate with actual treatment response. Similarly, Hashmi et al. showed that the transition of back pain patients from an acute- to a longer-term chronic-pain phenotype paralleled the evolution of resting-state abnormalities from acute-pain “sensory” to “emotion-related” brain regions (21). Thus, it would appear that a longer-duration of TN—itself related to poorer surgical outcome—is associated with limbic system changes increasing treatment-resistance, and rendering less effective any peripheral surgical treatments aimed at CNV. In turn, this would argue in favor of earlier surgical intervention in TN, which has been suggested to produce more durable pain relief (37). Furthermore, the functional networks identified in the present study may serve as potential pre-operative biomarkers of surgical outcome for TN and may also represent potential neurosurgical targets for TN or other pain conditions (38–40). We are currently carrying out further studies in which functional connectivity of the limbic system is compared between short-duration and longer-duration TN patients, and longitudinal studies in which functional connectivity is evaluated before and after TN surgery, in order to better understand the impact of limbic networks on treatment-resistance, the utility of limbic network connectivity as a biomarker of surgical outcome, and the extent to which altered limbic networks can actually be normalized by surgical intervention.

## Limitations

This study is not without limitations, most notably the small sample size with relatively few non-responders. We aimed to mitigate this limitation to some extent with a hypothesis-driven approach in which the analysis was restricted to only six pain-relevant brain structures chosen *a priori*. Our focused approach, however, limited our capacity to identify other brain regions whose function may also influence surgical response; larger sample studies with the statistical freedom to evaluate the whole-brain will allows us to replicate, and build on, our findings here. The small size and unbalanced nature of our cohort also prohibited receiver-operator characteristic (ROC) curve analysis to evaluate the sensitivity and specificity of functional connectivity in classifying surgical responders and non-responders (41, 42). To illustrate another limitation, it is worth pointing out that one patient in the non-responder group reported a VAS pain score of only 2/100 in the week preceding MRI scanning as they were experiencing a short period of remission. This patient has since been treated surgically three additional times—one of the most severe treatment-resistant cases of TN in our cohort—illustrating the challenge of accurately measuring pain severity in a fluctuating disease with periods of remission. To at least partially mitigate this limitation, we did adjust for pre-scan VAS as highlighted in the Methods. While medication class use did not differ between responders and non-responders at the group level, it must be pointed out that 50% of responders were exclusively taking first-line medications for their TN at the time of scanning. Therefore, we cannot rule out the possibility that the collective addition of second- and third-line medications in non-responders could be contributing to between-group connectivity differences. Another possible limitation is that we included a small number of patients undergoing repeat surgical treatments, in which functional connectivity may have been altered by prior surgery. That being said, it is noteworthy that repeat surgery patients were not distinguishable from virgin patients by any specific clinical attributes, nor did the proportion of repeat patients differ between response groups. Finally, the binarization of response to surgical treatment for TN (i.e., responder vs. non-responder) is an oversimplification, though common practice in the TN literature.

## Conclusion

We report a novel functional connectivity analysis in patients with TN undergoing surgical treatment. As in other chronic pain conditions, functional abnormalities in sensory-salience regions are also present in patients with TN. However, alterations in functional connectivity within limbic networks—which are correlated with increasing duration of illness—may be associated with the development of treatment-resistant pain that responds more poorly to surgery in certain patients with TN.

## Data Availability Statement

The raw data supporting the conclusions of this article will be made available by the authors, without undue reservation.

## Ethics Statement

The studies involving human participants were reviewed and approved by University of Alberta Health Research Ethics Board—Health Panel. The patients/participants provided their written informed consent to participate in this study. Written informed consent was obtained from the individual(s) for the publication of any potentially identifiable images or data included in this article.

## Author Contributions

HD designed the project, was responsible for all patient data collection, contributed to ethics approval, performed data analysis and results interpretation, and wrote the manuscript. SL contributed to data analysis, results interpretation, and wrote parts of the methods section. OM contributed to results interpretation and manuscript generation. TS oversaw all aspects of the study and directly contributed to study design, ethics approval, data analysis, results interpretation, and manuscript generation. All authors reviewed the manuscript and agreed to its submission.

## Funding

This work was funded by University of Alberta Hospital Foundation Sankar Research Establishment Grant, Edmonton Civic Employees Charitable Assistance Fund, Backman Family Fund (TS). Canada Research Chair in Non-Motor Symptoms of Parkinson's disease and the Tourmaline Chair in Parkinson's disease (OM).

## Conflict of Interest

The authors declare that the research was conducted in the absence of any commercial or financial relationships that could be construed as a potential conflict of interest.

## Publisher's Note

All claims expressed in this article are solely those of the authors and do not necessarily represent those of their affiliated organizations, or those of the publisher, the editors and the reviewers. Any product that may be evaluated in this article, or claim that may be made by its manufacturer, is not guaranteed or endorsed by the publisher.

## Abbreviations

TN: trigeminal neuralgia
CNV: cranial nerve V
MVD: microvascular decompression
MRI: magnetic resonance imaging
ACC: anterior cingulate cortex
fMRI: functional magnetic resonance imaging
PCC: posterior cingulate cortex
ICHD-III: International Classification of Headache Disorders-III
BC: percutaneous balloon compression rhizotomy
HC: healthy control
VAS: visual analog scale
ROI: region of interest
ROC: receiver operator characteristic.

## References

[1] Olesen J. Headache classification committee of the international headache society (IHS) the international classification of headache disorders, 3rd edition. Cephalalgia. (2018) 38:1–211. 10.1177/0333102417738202 29368949

[2] Zakrzewska JM Akram H. Neurosurgical interventions for the treatment of classical trigeminal neuralgia. Cochrane Database Syst Rev. (2011). 9:CD007312. 10.1002/14651858.CD007312.pub2 21901707 PMC8981212

[3] Obermann M. Treatment options in trigeminal neuralgia. Ther Adv Neurol Disord. (2010) 3:107–15. 10.1177/1756285609359317 21179603 PMC3002644

[4] Burchiel KIMJ Clarke H Haglund M Loeser J. Long-term efficacy of microvascular decompression in trigeminal neuralgia. J Neurosurg. (1988) 69:35–8. 10.3171/jns.1988.69.1.0035 2454303

[5] Desouza DD Hodaie M Davis KD. Abnormal trigeminal nerve microstructure and brain white matter in idiopathic trigeminal neuralgia. Pain. (2014) 155:37–44. 10.1016/j.pain.2013.08.029 23999058

[6] Lutz J Thon N Stahl R Lummel N Tonn J-C Linn J. Microstructural alterations in trigeminal neuralgia determined by diffusion tensor imaging are independent of symptom duration, severity, and type of neurovascular conflict. J Neurosurg. (2015) 124:823–30. 10.3171/2015.2.JNS142587 26406792

[7] DeSouza DD Davis KD Hodaie M. Reversal of insular and microstructural nerve abnormalities following effective surgical treatment for trigeminal neuralgia. Pain. (2015) 156:1112–23. 10.1097/j.pain.0000000000000156 25782366

[8] Tsai YH Yuan R Patel D Chandrasekaran S Weng HH Yang JT. Altered structure and functional connection in patients with classical trigeminal neuralgia. Hum Brain Mapp. (2018) 39:609–21. 10.1002/hbm.23696 29105886 PMC6866571

[9] Danyluk H Lee EK Wong S Sajida S Broad R Wheatley M. Hippocampal and trigeminal nerve volume predict outcome of surgical treatment for trigeminal neuralgia. Cephalalgia. (2020) 40:586–96. 10.1177/0333102419877659 31752520

[10] Zhang Y Mao Z Pan L Ling Z Liu X Zhang J. Dysregulation of pain- and emotion-related networks in trigeminal neuralgia. Front Hum Neurosci. (2018) 12:107. 10.3389/fnhum.2018.00107 29662445 PMC5890150

[11] Seeley WW Menon V Schatzberg AF Keller J Glover GH Kenna H. Dissociable intrinsic connectivity networks for salience processing and executive control. J Neurosci. (2007) 27:2349–56. 10.1523/JNEUROSCI.5587-06.2007 17329432 PMC2680293

[12] Wang Y Cao DY Remeniuk B Krimmel S Seminowicz DA Zhang M. Altered brain structure and function associated with sensory and affective components of classic trigeminal neuralgia. Pain. (2017) 158:1561–70. 10.1097/j.pain.0000000000000951 28520647

[13] Henssen D Dijk J Knepflé R Sieffers M Winter A Vissers K. Alterations in grey matter density and functional connectivity in trigeminal neuropathic pain and trigeminal neuralgia: a systematic review and meta-analysis. NeuroImage Clin. (2019) 24:102039. Available from: 10.1016/j.nicl.2019.102039 31698316 PMC6978224

[14] Obermann M Yoon MS Ese D Maschke M Kaube H Diener HC. Impaired trigeminal nociceptive processing in patients with trigeminal neuralgia. Neurology. (2007) 69:835–41. 10.1212/01.wnl.0000269670.30045.6b 17724285

[15] Rodriguez-Raecke R Niemeier A Ihle K Ruether W May A. Brain gray matter decrease in chronic pain is the consequence and not the cause of pain. J Neurosci. (2009) 29:13746–50. 10.1523/JNEUROSCI.3687-09.2009 19889986 PMC6666725

[16] (Bud) Craig AD. How do you feel — now? The anterior insula and human awareness. Nat Rev Neurosci. (2009) 10:59–70. 10.1038/nrn2555 19096369

[17] Shackman AJ Salomons T V Slagter HA Fox AS Winter JJ Davidson RJ. The integration of negative affect, pain and cognitive control in the cingulate cortex. Nat Rev Neurosci. (2011) 12:154–67. 10.1038/nrn2994 21331082 PMC3044650

[18] Tso AR Trujillo A Guo CC Goadsby PJ Seeley WW. The anterior insula shows heightened interictal intrinsic connectivity in migraine without aura. Neurology. (2015) 84:1043–50. 10.1212/WNL.0000000000001330 25663219 PMC4352101

[19] Lickteig R Lotze M Kordass B. Successful therapy for temporomandibular pain alters anterior insula and cerebellar representations of occlusion. Cephalalgia. (2013) 33:1248–57. 10.1177/0333102413491028 23771211

[20] Yarkoni T Poldrack RA Nichols TE Van Essen DC Wager TD. Large-scale automated synthesis of human functional neuroimaging data. Nat Methods. (2011) 8:665–70. 10.1038/nmeth.1635 21706013 PMC3146590

[21] Hashmi JA Baliki MN Huang L Baria AT Torbey S Hermann KM. Shape shifting pain: chronification of back pain shifts brain representation from nociceptive to emotional circuits. Brain. (2013) 136:2751–68. 10.1093/brain/awt211 23983029 PMC3754458

[22] Heinskou TB Rochat P Maarbjerg S Wolfram F Brennum J Olesen J. Prognostic factors for outcome of microvascular decompression in trigeminal neuralgia: a prospective systematic study using independent assessors. Cephalalgia. (2019) 39:197–208. 10.1177/0333102418783294 29896973

[23] Han PP Shetter AG Smith KA Fiedler JA Rogers CL Speiser B. Gamma knife radiosurgery for trigeminal neuralgia: experience at the barrow neurological institute. Int J Radiat Oncol Biol Phys. (2000) 47:1013–9. 10.1016/S0360-3016(00)00513-7 10863073

[24] Ashburner J Friston K. Unified segmentation. Neuroimage. (2005) 26:839–51. 10.1016/j.neuroimage.2005.02.018 15955494

[25] Calhoun V Wager T Krishan A Rosch K Seymour K Nebel M. The impact of T1 versus EPI spatial normalization templates for fMRI data analyses. Human. (2017) 38:5331–42. 10.1002/hbm.23737 28745021 PMC5565844

[26] Whitfield-Gabrieli S Nieto-Castanon A. Conn: a functional connectivity toolbox for correlated and anticorrelated brain networks. Brain Connect. (2012) 2:125–41. 10.1089/brain.2012.0073 22642651

[27] Behzadi Y Restom K Liu T. A component based noise correction method for BOLD and perfusion based fMRI. Neuroimage. (2007) 37:90–101. 10.1016/j.neuroimage.2007.04.042 17560126 PMC2214855

[28] Desikan RS Ségonne F Fischl B Quinn BT Dickerson BC Blacker D. An automated labeling system for subdividing the human cerebral cortex on MRI scans into gyral based regions of interest. Neuroimage. (2006) 31:968–80. 10.1016/j.neuroimage.2006.01.021 16530430

[29] Lim M Jassar H Kim DJ Nascimento TD DaSilva AF. Differential alteration of fMRI signal variability in the ascending trigeminal somatosensory and pain modulatory pathways in migraine. J Headache Pain. (2021) 22:4. 10.1186/s10194-020-01210-6 33413090 PMC7791681

[30] Danyluk H Andrews J Kesarwani R Seres P Broad R Wheatley BM. The thalamus in trigeminal neuralgia : structural and metabolic abnormalities, and influence on surgical response. BMC Neurol. (2021) 21:290. 10.1186/s12883-021-02323-4 34303364 PMC8305513

[31] Kong J White NS Kwong KK Vangel MG Rosman IS Gracely RH. Using fMRI to dissociate sensory encoding from cognitive evaluation of heat pain intensity. Hum Brain Mapp. (2006) 27:715–21. 10.1002/hbm.20213 16342273 PMC6871429

[32] Ghaziri J Tucholka A Girard G Boucher O Houde JC Descoteaux M. Subcortical structural connectivity of insular subregions. Sci Rep. (2018) 8:8596. 10.1038/s41598-018-26995-0 29872212 PMC5988839

[33] Wang Y Yang Q Cao D Seminowicz D Remeniuk B Gao L. Correlation between nerve atrophy, brain grey matter volume and pain severity in patients with primary trigeminal neuralgia. Cephalalgia. (2018) 39:515–25. 10.1177/0333102418793643 30086682 PMC8889450

[34] Apkarian AV Mutso AA Centeno MV Kan L Wu M Levinstein M. Role of adult hippocampal neurogenesis in persistent pain. Pain. (2016) 157:418–28. 10.1097/j.pain.0000000000000332 26313405 PMC4858177

[35] Berger SE Vachon-Presseau É Abdullah TB Baria AT Schnitzer TJ Apkarian AV. Hippocampal morphology mediates biased memories of chronic pain. Neuroimage. (2018) 166:86–98. 10.1016/j.neuroimage.2017.10.030 29080714 PMC5813825

[36] Shimo K Ueno T Younger J Nishihara M Inoue S Ikemoto T. Visualization of painful experiences believed to trigger the activation of affective and emotional brain regions in subjects with low back pain. PLoS ONE. (2011) 6:2–7. 10.1371/journal.pone.0026681 22073183 PMC3206847

[37] Mousavi SH Niranjan A Huang MJ Laghari FJ Shin SS Mindlin JL. Early radiosurgery provides superior pain relief for trigeminal neuralgia patients. Neurology. (2015) 85:2159–65. 10.1212/WNL.0000000000002216 26561286

[38] Russo JF Sheth SA. Deep brain stimulation of the dorsal anterior cingulate cortex for the treatment of chronic neuropathic pain. Neurosurg Focus. (2015) 38:E11. 10.3171/2015.3.FOCUS1543 26030699

[39] Yen CP Kung SS Su YF Lin WC Howng SL Kwan AL. Stereotactic bilateral anterior cingulotomy for intractable pain. J Clin Neurosci. (2005) 12:886–90. 10.1016/j.jocn.2004.11.018 16326270

[40] Gallay MN Moser D Jeanmonod D. MR-Guided focused ultrasound central lateral thalamotomy for trigeminal neuralgia. Single center experience. Front Neurol. (2020) 11:271. 10.3389/fneur.2020.00271 32425870 PMC7212452

[41] Saito T Rehmsmeier M. The precision-recall plot is more informative than the ROC plot when evaluating binary classifiers on imbalanced datasets. PLoS ONE. (2015) 10:e0118432. 10.1371/journal.pone.0118432 25738806 PMC4349800

[42] Fawcett T. An introduction to ROC analysis. Patt Recog. Lett. (2006) 27:861–74. 10.1016/j.patrec.2005.10.010

