# Supplementary material for: Pre-operative Limbic System Functional Connectivity Distinguishes Responders From Non-responders to Surgical Treatment for Trigeminal Neuralgia
Source: Front Neurol. 2021 Oct 4;12:716500. doi: 10.3389/fneur.2021.716500 (PMC8520903; doi:10.3389/fneur.2021.716500)
Supplement: Supplementary file 1 [file Table_1.DOCX]

**SUPPLEMENTAL MATERIAL (1 table, 2 figures)**

**A) Average z-transformed correlation coefficient (Healthy control)**

|  | IC r | IC l | Am r | Am l | Th r | Th l | ACC | PCC | Hp r | Hp l |
| --- | --- | --- | --- | --- | --- | --- | --- | --- | --- | --- |
| IC r | NaN | 0.904 | 0.073 | 0.113 | 0.180 | 0.112 | 0.386 | 0.010 | 0.057 | 0.032 |
| IC l | 0.904 | NaN | 0.028 | 0.089 | 0.177 | 0.178 | 0.364 | 0.012 | 0.033 | 0.053 |
| Am r | 0.073 | 0.028 | NaN | 0.386 | 0.086 | 0.064 | 0.072 | 0.066 | 0.330 | 0.244 |
| Am l | 0.113 | 0.089 | 0.386 | NaN | 0.084 | 0.108 | 0.089 | 0.056 | 0.311 | 0.322 |
| Th r | 0.180 | 0.177 | 0.086 | 0.084 | NaN | 0.672 | 0.195 | 0.200 | 0.083 | 0.072 |
| Th l | 0.112 | 0.178 | 0.064 | 0.108 | 0.672 | NaN | 0.182 | 0.226 | 0.072 | 0.098 |
| AC | 0.386 | 0.364 | 0.072 | 0.089 | 0.195 | 0.182 | NaN | 0.464 | 0.089 | 0.094 |
| PC | 0.010 | 0.012 | 0.066 | 0.056 | 0.200 | 0.226 | 0.464 | NaN | 0.156 | 0.171 |
| Hp r | 0.057 | 0.033 | 0.330 | 0.311 | 0.083 | 0.072 | 0.089 | 0.156 | NaN | 0.445 |
| Hp l | 0.032 | 0.053 | 0.244 | 0.322 | 0.072 | 0.098 | 0.094 | 0.171 | 0.445 | NaN |

**B) Average z-transformed correlation coefficient (TN)**

|  | IC r | IC l | Am r | Am l | Th r | Th l | ACC | PCC | Hp r | Hp l |
| --- | --- | --- | --- | --- | --- | --- | --- | --- | --- | --- |
| IC r | NaN | 0.907 | 0.087 | 0.064 | 0.331 | 0.282 | 0.316 | 0.094 | 0.086 | 0.053 |
| IC l | 0.907 | NaN | 0.036 | 0.075 | 0.282 | 0.278 | 0.307 | 0.084 | 0.064 | 0.088 |
| Am r | 0.087 | 0.036 | NaN | 0.385 | 0.128 | 0.112 | 0.020 | 0.045 | 0.429 | 0.330 |
| Am l | 0.064 | 0.075 | 0.385 | NaN | 0.074 | 0.084 | 0.061 | 0.069 | 0.343 | 0.390 |
| Th r | 0.331 | 0.282 | 0.128 | 0.074 | NaN | 0.804 | 0.096 | 0.136 | 0.101 | 0.079 |
| Th l | 0.282 | 0.278 | 0.112 | 0.084 | 0.804 | NaN | 0.103 | 0.159 | 0.077 | 0.107 |
| AC | 0.316 | 0.307 | 0.020 | 0.061 | 0.096 | 0.103 | NaN | 0.418 | 0.015 | 0.000 |
| PC | 0.094 | 0.084 | 0.045 | 0.069 | 0.136 | 0.159 | 0.418 | NaN | 0.131 | 0.098 |
| Hp r | 0.086 | 0.064 | 0.429 | 0.343 | 0.101 | 0.077 | 0.015 | 0.131 | NaN | 0.518 |
| Hp l | 0.053 | 0.088 | 0.330 | 0.390 | 0.079 | 0.107 | 0.000 | 0.098 | 0.518 | NaN |

**C) Average z-transformed correlation coefficient (Responder)**

|  | IC r | IC l | Am r | Am l | Th r | Th l | ACC | PCC | Hp r | Hp l |
| --- | --- | --- | --- | --- | --- | --- | --- | --- | --- | --- |
| IC r | NaN | 0.838 | 0.130 | 0.098 | 0.296 | 0.243 | 0.366 | 0.139 | 0.108 | 0.083 |
| IC l | 0.838 | NaN | 0.077 | 0.091 | 0.249 | 0.240 | 0.350 | 0.126 | 0.084 | 0.104 |
| Am r | 0.130 | 0.077 | NaN | 0.335 | 0.140 | 0.115 | 0.055 | 0.069 | 0.389 | 0.292 |
| Am l | 0.098 | 0.091 | 0.335 | NaN | 0.080 | 0.079 | 0.093 | 0.098 | 0.308 | 0.332 |
| Th r | 0.296 | 0.249 | 0.140 | 0.080 | NaN | 0.735 | 0.129 | 0.151 | 0.113 | 0.091 |
| Th l | 0.243 | 0.240 | 0.115 | 0.079 | 0.735 | NaN | 0.141 | 0.183 | 0.084 | 0.120 |
| AC | 0.366 | 0.350 | 0.055 | 0.093 | 0.129 | 0.141 | NaN | 0.409 | 0.050 | 0.026 |
| PC | 0.139 | 0.126 | 0.069 | 0.098 | 0.151 | 0.183 | 0.409 | NaN | 0.154 | 0.108 |
| Hp r | 0.108 | 0.084 | 0.389 | 0.308 | 0.113 | 0.084 | 0.050 | 0.154 | NaN | 0.438 |
| Hp l | 0.083 | 0.104 | 0.292 | 0.332 | 0.091 | 0.120 | 0.026 | 0.108 | 0.438 | NaN |

**D) Average z-transformed correlation coefficient (Non-Responder)**

|  | IC r | IC l | Am r | Am l | Th r | Th l | ACC | PCC | Hp r | Hp l |
| --- | --- | --- | --- | --- | --- | --- | --- | --- | --- | --- |
| IC r | NaN | 1.091 | -0.029 | -0.027 | 0.423 | 0.386 | 0.183 | -0.027 | 0.026 | -0.026 |
| IC l | 1.091 | NaN | -0.073 | 0.031 | 0.370 | 0.377 | 0.193 | -0.028 | 0.009 | 0.043 |
| Am r | -0.029 | -0.073 | NaN | 0.520 | 0.096 | 0.105 | -0.074 | -0.021 | 0.537 | 0.429 |
| Am l | -0.027 | 0.031 | 0.520 | NaN | 0.059 | 0.097 | -0.024 | -0.008 | 0.436 | 0.543 |
| Th r | 0.423 | 0.370 | 0.096 | 0.059 | NaN | 0.988 | 0.008 | 0.095 | 0.070 | 0.048 |
| Th l | 0.386 | 0.377 | 0.105 | 0.097 | 0.988 | NaN | 0.001 | 0.097 | 0.059 | 0.071 |
| AC | 0.183 | 0.193 | -0.074 | -0.024 | 0.008 | 0.001 | NaN | 0.443 | -0.078 | -0.070 |
| PC | -0.027 | -0.028 | -0.021 | -0.008 | 0.095 | 0.097 | 0.443 | NaN | 0.069 | 0.072 |
| Hp r | 0.026 | 0.009 | 0.537 | 0.436 | 0.070 | 0.059 | -0.078 | 0.069 | NaN | 0.731 |
| Hp l | -0.026 | 0.043 | 0.429 | 0.543 | 0.048 | 0.071 | -0.070 | 0.072 | 0.731 | NaN |

**E) P-value Table (Healthy Control vs TN)**

|  | IC r | IC l | Am r | Am l | Th r | Th l | ACC | PCC | Hp r | Hp l |
| --- | --- | --- | --- | --- | --- | --- | --- | --- | --- | --- |
| IC r | NaN | 0.968 | 0.802 | 0.345 | **0.002** | **0.0007** | 0.348 | 0.086 | 0.592 | 0.595 |
| IC l | 0.968 | NaN | 0.871 | 0.77 | 0.030 | 0.051 | 0.45 | 0.098 | 0.559 | 0.424 |
| Am r | 0.802 | 0.871 | NaN | 0.996 | 0.355 | 0.342 | 0.232 | 0.548 | 0.047 | 0.077 |
| Am l | 0.345 | 0.77 | 0.996 | NaN | 0.812 | 0.605 | 0.429 | 0.735 | 0.462 | 0.239 |
| Th r | **0.002** | 0.030 | 0.355 | 0.812 | NaN | 0.103 | 0.066 | 0.081 | 0.656 | 0.874 |
| Th l | **0.0007** | 0.051 | 0.342 | 0.605 | 0.103 | NaN | 0.115 | 0.083 | 0.89 | 0.841 |
| AC | 0.348 | 0.45 | 0.232 | 0.429 | 0.066 | 0.115 | NaN | 0.418 | 0.088 | 0.037 |
| PC | 0.086 | 0.098 | 0.548 | 0.735 | 0.081 | 0.083 | 0.418 | NaN | 0.629 | 0.161 |
| Hp r | 0.592 | 0.559 | 0.047 | 0.462 | 0.656 | 0.89 | 0.088 | 0.629 | NaN | 0.284 |
| Hp l | 0.595 | 0.424 | 0.077 | 0.239 | 0.874 | 0.841 | 0.037 | 0.161 | 0.284 | NaN |

**F) P-value table (Responder vs non-Responder)**

|  | IC r | IC l | Am r | Am l | Th r | Th l | ACC | PCC | Hp r | Hp l |
| --- | --- | --- | --- | --- | --- | --- | --- | --- | --- | --- |
| IC r | NaN | 0.194 | 0.14 | 0.161 | 0.046 | 0.066 | 0.156 | 0.036 | 0.426 | 0.07 |
| IC l | 0.194 | NaN | 0.115 | 0.577 | 0.097 | 0.109 | 0.186 | 0.029 | 0.488 | 0.403 |
| Am r | 0.14 | 0.115 | NaN | 0.115 | 0.7 | 0.995 | 0.017 | 0.076 | 0.106 | 0.061 |
| Am l | 0.161 | 0.577 | 0.115 | NaN | 0.907 | 0.697 | **0.008** | 0.14 | 0.103 | 0.021 |
| Th r | 0.046 | 0.097 | 0.7 | 0.907 | NaN | 0.043 | 0.157 | 0.277 | 0.67 | 0.596 |
| Th l | 0.066 | 0.109 | 0.995 | 0.697 | 0.043 | NaN | 0.065 | 0.082 | 0.816 | 0.519 |
| AC | 0.156 | 0.186 | 0.017 | **0.008** | 0.157 | 0.065 | NaN | 0.6 | **0.005** | 0.062 |
| PC | 0.036 | 0.029 | 0.076 | 0.14 | 0.277 | 0.082 | 0.6 | NaN | 0.197 | 0.656 |
| Hp r | 0.426 | 0.488 | 0.106 | 0.103 | 0.67 | 0.816 | **0.005** | 0.197 | NaN | **0.011** |
| Hp l | 0.07 | 0.403 | 0.061 | 0.021 | 0.596 | 0.519 | 0.062 | 0.656 | **0.011** | NaN |

**Supplemental Table 1:** Raw functional connectivity results between for TN patients and healthy control subjects (**A-F**). **IC r** = insular cortex right; **IC l** = insular cortex left; **Am r** = amygdala right; **Am l** = amygdala left; **Th r** = thalamus right; **Th l** = thalamus left; **ACC** = anterior cingulate; **PCC** = posterior cingulate; **Hp r** = hippocampus right; **Hp l** = hippocampus left. Uncorrected p-values are displayed. Bold typeface = significant result after FDR correction.

**Supplemental Figure 1**: Peri-operative TN pain severity. TN pain is described using the visual analogue pain scale (VAS) score for TN patients at pre-operative (<1-month before surgery), immediate post-operative (<1-month after surgery), and pain recurrence time-points. Responders and non-responders are indicated with green circles and red triangles respectively. **Pre-op**: pre-operative; **Post-op (immed)**: immediate post-operative; **Rec.**: recurrence.

**Supplemental Figure 2**: Outcomes of surgical treatment for TN. Barrow Neurological Institute (BNI) pain intensity score over a 1-year post-operative period is reported for responders (**A**—BNI 1, 2, 3a) and non-responders (**B**—BNI 3b, 4, and 5). Note: there were no patients with BNI score 3b.
